# Ablation of Matrix Metalloproteinase-9 Prevents Cardiomyocytes Contractile Dysfunction in Diabetics

**DOI:** 10.3389/fphys.2016.00093

**Published:** 2016-03-15

**Authors:** Priyanka Prathipati, Naira Metreveli, Shyam Sundar Nandi, Suresh C. Tyagi, Paras K. Mishra

**Affiliations:** ^1^Department of Cellular and Integrative Physiology, University of Nebraska Medical CenterOmaha, NE, USA; ^2^Department of Physiology and Biophysics, University of LouisvilleLouisville, KY, USA; ^3^Department of Anesthesiology, University of Nebraska Medical CenterOmaha, NE, USA

**Keywords:** heart failure, SERCA-2a, calcium transient, Akita, diabetes

## Abstract

Elevated expression and activity of matrix metalloproteinase-9 (MMP9) and decreased contractility of cardiomyocytes are documented in diabetic hearts. However, it is unclear whether MMP is involved in the regulation of contractility of cardiomyocytes in diabetic hearts. In the present study, we tested the hypothesis that MMP9 regulates contractility of cardiomyocytes in diabetic hearts, and ablation of MMP9 prevents impaired contractility of cardiomyocytes in diabetic hearts. To determine the specific role of MMP9 in cardiomyocyte contractility, we used 12–14 week male WT (normoglycemic sibling of Akita), Akita, and Ins^2+∕−^/MMP9^−∕−^ (DKO) mice. DKO mice were generated by cross-breeding male Ins2^+∕−^ Akita (T1D) with female MMP9 knockout (MMP9^−∕−^) mice. We isolated cardiomyocytes from the heart of the above three groups of mice and measured their contractility and calcium transients. Moreover, we determined mRNA and protein levels of sarco-endoplasmic reticulum calcium ATPase-2a (SERCA-2a), which is involved in calcium handling during contractility of cardiomyocytes in WT, Akita, and DKO hearts using QPCR, Western blotting and immunoprecipitation, respectively. Our results revealed that in Akita hearts where increased expression and activity of MMP9 is reported, the rates of shortening and re-lengthening (±dL/dt) of cardiomyocytes were decreased, time to 90% peak height and baseline during contractility was increased, rate of calcium decay was increased, and calcium transient was decreased as compared to WT cardiomyocytes. However, these changes in Akita were blunted in DKO cardiomyocytes. The molecular analyses of SERCA-2a in the hearts showed that it was downregulated in Akita as compared to WT but was comparatively upregulated in DKO. These results suggest that abrogation of MMP9 gene prevents contractility of cardiomyocytes, possibly by increasing SERCA-2a and calcium transients. We conclude that MMP9 plays a crucial role in the regulation of contractility of cardiomyocytes in diabetic hearts.

## Introduction

Impaired contractility is a hallmark of all forms of heart failure including diabetic heart failure (Mishra et al., 2010a; Luo and Anderson, 2013). Diabetic cardiomyopathy is independent of ischemia, valvular disease, or hypertension (Rubler et al., 1972). In diabetics, the heart initially undergoes diastolic dysfunction, which at advanced stage progresses to systolic dysfunction (Boudina and Abel, 2007). Decreased contractility of cardiomyocytes has an important role in cardiac dysfunction, and calcium plays a crucial role in cardiomyocyte contractility. The sarcoplasmic endoplasmic reticulum calcium ATPase (SERCA-2a) is a calcium handling protein (Giordano et al., 1997; He et al., 1997; del Monte et al., 1999; ter Keurs, 2012; Luo and Anderson, 2013). In diabetic hearts SERCA-2a is downregulated and contractility of cardiomyocytes is impaired (Mishra et al., 2010a,b). On the other hand, expression and activity of matrix metalloproteinase-9 (MMP9), a Zn^2+^-dependent collagenase, is increased in diabetic hearts (Mishra et al., 2010b). MMP9 is demonstrated to reduce contractility of non-diabetic cardiomyocytes (Mishra et al., 2010c). However, the role of MMP9 in cardiomyocytes contractility in diabetics is unclear. To determine the role of MMP9 in cardiomyocyte contractility, we used Insulin2 mutant (Ins2^+∕−^) Akita mice. Akita is a genetic, spontaneous, and chronic model of type1 diabetes (T1D), which is relevant to humans (Insulin 2 gene of mouse is orthologous to human Insulin gene), and where mutation of Insulin gene causes T1D (Chavali et al., 2014a). In Akita mice, MMP9 is activated in the hearts (Mishra et al., 2010b) and contractility of cardiomyocytes is decreased (Mishra et al., 2010a). To assess the specific role of MMP9 on contractility of cardiomyocytes in diabetic Akita, we generated Ins2^+∕−^/MMP9^−∕−^ (DKO) mice, which are diabetic but without MMP9 gene. We have validated by genotyping, Western blotting, zymography, and confocal microscopy that 12–14 week male DKO mice have MMP9 gene deleted and MMP9 activity nullified in the heart (Mishra et al., 2012). In the present study, we used DKO mice to determine the specific role of MMP9 on cardiomyocytes contractility in diabetics.

## Methods

### Animal models

Male C57BL/6J, MMP9^−∕−^ and diabetic Ins2^+∕−^ Akita mice were procured from the Jackson Laboratories (Bar Harbor, ME, USA). They were maintained in the animal facility of the University of Louisville (Louisville, KY) under a 12:12 h light-dark cycle. Standard diet and water *ad libitum* was provided to the mice. DKO mice were generated by cross-breeding male Ins2^+∕−^ Akita with female MMP9^−∕−^ mice as described elsewhere (Mishra et al., 2012; Chavali et al., 2014b). Twelve-fourteen week male mice were used in all experiments. Animal cross-breeding, extraction of the heart, and isolation of cardiomyocytes were performed at the University of Louisville following protocols approved by the Institutional Animal Care and Use Committee of the University of Louisville.

### Experimental methods

#### Isolation of ventricular cardiomyocytes

Cardiomyocytes were isolated from the left ventricle by the enzymatic dissociation as described elsewhere (Mishra et al., 2010c). In brief, the heart was excised and perfused with calcium free perfusion buffer until they get soft and limp. Subsequently, it was perfused with digestion buffer. The left ventricle was removed, minced under sterile conditions, and suspended in perfusion buffer to dissociate cardiomyocytes. These cardiomyocytes were kept in contractility buffer (135 mM NaCl, 4 mM KCl, 1 mM MgCl_2_, 10 mM HEPES, 0.33 mM NaH_2_PO_4_, 10 mM glucose, 10 mM BDM, 1.2 mM CaCl_2_). Cardiomyocytes were gently pipetted up and down with a plastic pipette (2 mm tip) several times. Then the cells were transferred to a 15 ml sterile polypropylene conical tube where 10, 20, 30, 40, and 100 μl of a 30 mM CaCl_2_ solution was added at 5 min intervals. The final content of calcium was 1.2 mM. Isolated myocytes were maintained at room temperature in this buffer.

#### Determination of cell shortening and re-lengthening

Cardiomyocyte contractility was controlled by electrical stimulation. Mechanical properties of isolated ventricular myocytes were assessed by video-based edge detection. An inverted microscope, a low light-level video camera and a computer-based motion analyzer were used to track the movement of cell edges. The isolated myocytes were diluted approximately 10-fold with the contractility buffer and placed on a Teflon glass coverslip dish mounted on the stage of an inverted microscope (Olympus, IX-70). The cells were field stimulated to contract by the MyoPacer field stimulator through a pair of platinum electrodes at a frequency of 1.0 Hz, pulse duration of 4 ms and amplitude of 10 volts. The image of the myocyte was obtained with an IonOptix MyoCam camera side-mounted onto the microscope and displayed on a computer monitor using the Soft-Edge software. Typically five individual myocytes were recorded before replacing the myocytes with a fresh dilution of unstimulated myocytes. This prevented exhaustion of the myocytes due to prolonged stimulation. Twenty-twenty five myocytes were analyzed for each heart. The cells being studied were scanned every 8.3 ms so that the amplitude and velocity of shortening and lengthening can be recorded with good fidelity. The displacements of cell edges at both ends of the myocyte were detected and converted to an analog voltage signal, which was then digitized and stored for off-line analyses. Steady-state twitches (Malhotra and Sanghi, 1997; Mann and Spinale, 1998; Thomas et al., 1998; Tayebjee et al., 2004; Chu et al., 2013; Zarain-Herzberg et al., 2014) were analyzed for cell length changes using the Soft-Edge software and averaged for each myocyte. Cell shortening and relengthening were assessed by time to 90% peak shortening, time to 90% relengthening, and maximal velocities of shortening and relengthening (±dL/dt).

#### Intracellular calcium measurement

Intracellular Ca^2+^ influx was detected by using Fura 2-AM (Mishra et al., 2010c). Briefly, fluorescence was measured by loading the myocytes with 1.0 μmol/L of Fura 2-AM. Cells were illuminated at 360 nm for 0.5 s and then at 380 nm for the rest of the duration of recording. Fluorescence was detected between 480 and 520 nm using a photomultiplier tube during contraction/relaxation cycle. Intracellular Ca^2+^ clearance rate was determined from the fluorescence signal decay over the time. Ca^2+^ transients were analyzed in fifteen-twenty cardiomyocytes per animal and four- seven animals per group.

#### RNA extraction and quantitative PCR (QPCR)

RNA was extracted from the isolated hearts of WT, Akita, and DKO using Trizol kit (Thermo scientific, Waltham, MA, USA, cat # 15596-018) following the kit's protocol. The quality of the extracted RNA was determined by NanoDrop-2000 spectrophotometer. Pure quality RNA with 260/280~2.00 and 260/230~2.00 was used for further RT-PCR and quantitative-PCR analyses. The SERCA-2a primers were designed using Primer3 Input. The sequences for forward and reverse primers of SERCA-2a were 5′ TCTTCATAACACACGCCAATT 3′, and 5′ CCCTTTGCTGCCAATTAACTA 3′, respectively. The 18S control primer forward and reverse sequences were 5′ GTAGTTCCGACCATAAACGA 3′ and 5′ TCAATCTGTCAATCCTGTCC 3′, respectively. Syber-green dye (BioRad Laboratories, Hercules, CA, USA, catalog # 172-5120) was used for Real-Time quantitative PCR (QPCR). QPCR was performed on Bio-Rad CFX-connect system with gene amplification program: 95°C-3 min, [95°C-5 s, 55°C-15 s] × 39 as per the product protocol.

#### Protein extraction and western blotting

Tissues were lysed in RIPA buffer (Boston BioProducts, Ashland, MA, USA, cat # BP-115). BCA protein assay was used for protein quantification analyses with Pierce BCA protein assay kit (Thermo scientific, Waltham, MA, USA, cat # 23227). Absorbance was measured using Promega glow max multi + detection system (Promega, Madison, WI, USA). Equal amounts of protein (30 μg) were subjected to 10% SDS-PAGE and transferred to nitrocellulose membrane. Primary antibody used were: SERCA-2a (Abcam, Cambridge, MA, USA, cat # ab2861) and GAPDH (EMD Millipore, Billerica, MA, USA, cat # MAB 374). The secondary antibody was mouse (Santa Cruz Biotechnology, Dallas, TX, USA, cat # sc 2005). Clarity Western ECL substrate (BioRad Laboratories, Hercules, CA, USA, catalog # 170-5061) was used for developing the blots. Densitometric analysis was carried out using Image Lab software.

#### Immunoprecipitation

Immunoprecipitation was performed for SERCA-2a using Protein A dynabeads (Novex, Thermo scientific, Waltham, MA, USA, cat # 10001D) following the kit's protocol. Briefly, 4 μg of SERCA-2a antibody (Ab) was added to 50 μl of dynabeads. After incubating at room temperature for 10 min, beads were washed with 1x phosphate buffered saline (1xPBS) with Tween-20 (0.1%). Diluted heart tissue protein samples (400 μg in 500 μl of RIPA) (Ag) were added to the above beads and incubated for 30 min at room temperature. Dynabeads-Ab-Ag complex was then washed three times in 1xPBS. Bound complex was eluted using 2x sample buffer (BioRad Laboratories, Hercules, CA, USA, catalog # 161-0737) followed by 10 min heating at 95°C. Dissociated protein from the beads was collected and loaded onto the SDS-PAGE gel following the protocol of Western Blot. Proteins were transferred onto nitrocellulose membrane and probed by SERCA-2a antibody.

#### Statistical analysis

Data were expressed as mean ± SEM. One-way analysis of variance (ANOVA) and Tuckey's honest significant difference (HSD) test was used to determine the differences in means. *P* < 0.05 was considered significant and represented by “^*^”.

## Results

### Ablation of MMP9 gene restores cardiomyocyte contractility in diabetic hearts

**Figure 1:**
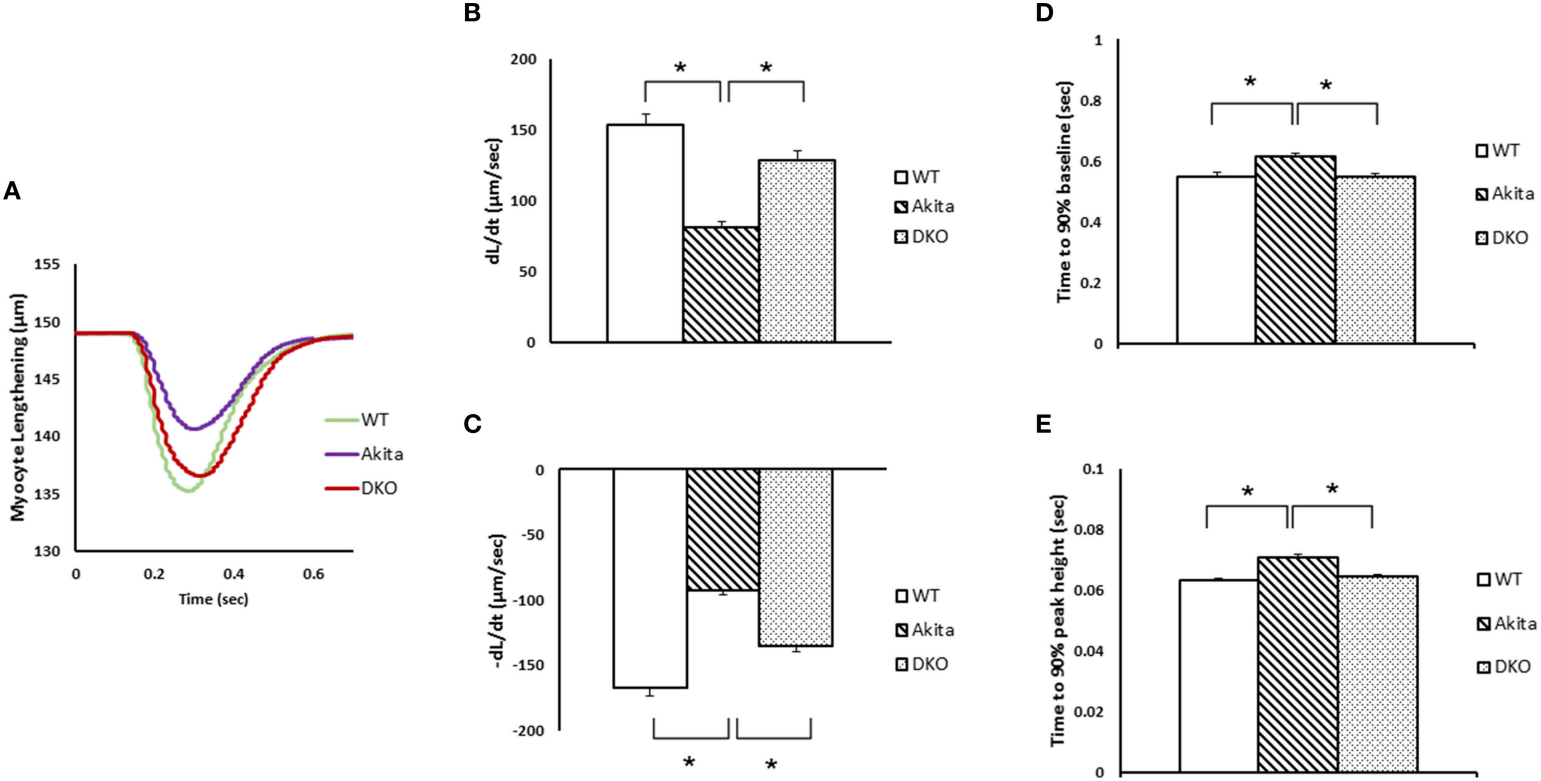
**Ablation of MMP9 prevents cardiomyocytes contractile dysfunction in diabetic Akita mice**. **(A)** Cardiomyocyte lengthening (μM) and duration of lengthening was measured in WT, Akita, and DKO hearts. Myocytes were stimulated at 1 Hz. **(B)** The rate of contraction (dL/dt), and **(C)** rate of relaxation (-dL/dt) was decreased in Akita but increased in DKO. **(D)** The time elapsed to attain 90% baseline, and **(E)** 90% peak height during contractility of cardiomyocytes was increased in Akita but restored in DKO. Values are mean ± SEM from 15 to 20 cardiomyocytes from each group. *N* = 4–7. ^*^p < 0.05.

We have reported earlier that the rates of contraction (dL/dt) and relaxation (-dL/dt) of cardiomyocytes are decreased (Mishra et al., 2010a), and cardiac MMP9 is activated (Mishra et al., 2010b) in diabetic Akita. To determine whether induction of MMP9 causes contractile dysfunction in diabetic hearts, we measured contractility of cardiomyocytes in WT, Akita, and DKO cardiomyocytes. Our results showed that myocyte lengthening was decreased in Akita as compared to WT but it was significantly improved in DKO cardiomyocytes (Figure 1A). The measurement of ±dL/dt showed that the rates of contraction and relaxation were decreased in Akita as compared to WT cardiomyocytes (Figures 1B,C). It was consistent with the previous report (Mishra et al., 2010a). In DKO cardiomyocytes ±dL/dt was significantly increased as compared to Akita cardiomyocytes (Figures 1B,C). Moreover, we measured the time required for attaining 90% peak height and 90% baseline to assess impaired contractility of cardiomyocytes. Our results demonstrated that Akita took significantly more time for 90% peak height and 90% baseline as compared to WT cardiomyocytes, however, it was restored in DKO (Figures 1D,E). These results suggest that ablation of MMP9 improves contractility of cardiomyocytes in diabetic hearts.

### Abrogation of MMP9 prolonged calcium transients in cardiomyocytes of DKO mice

**Figure 2:**
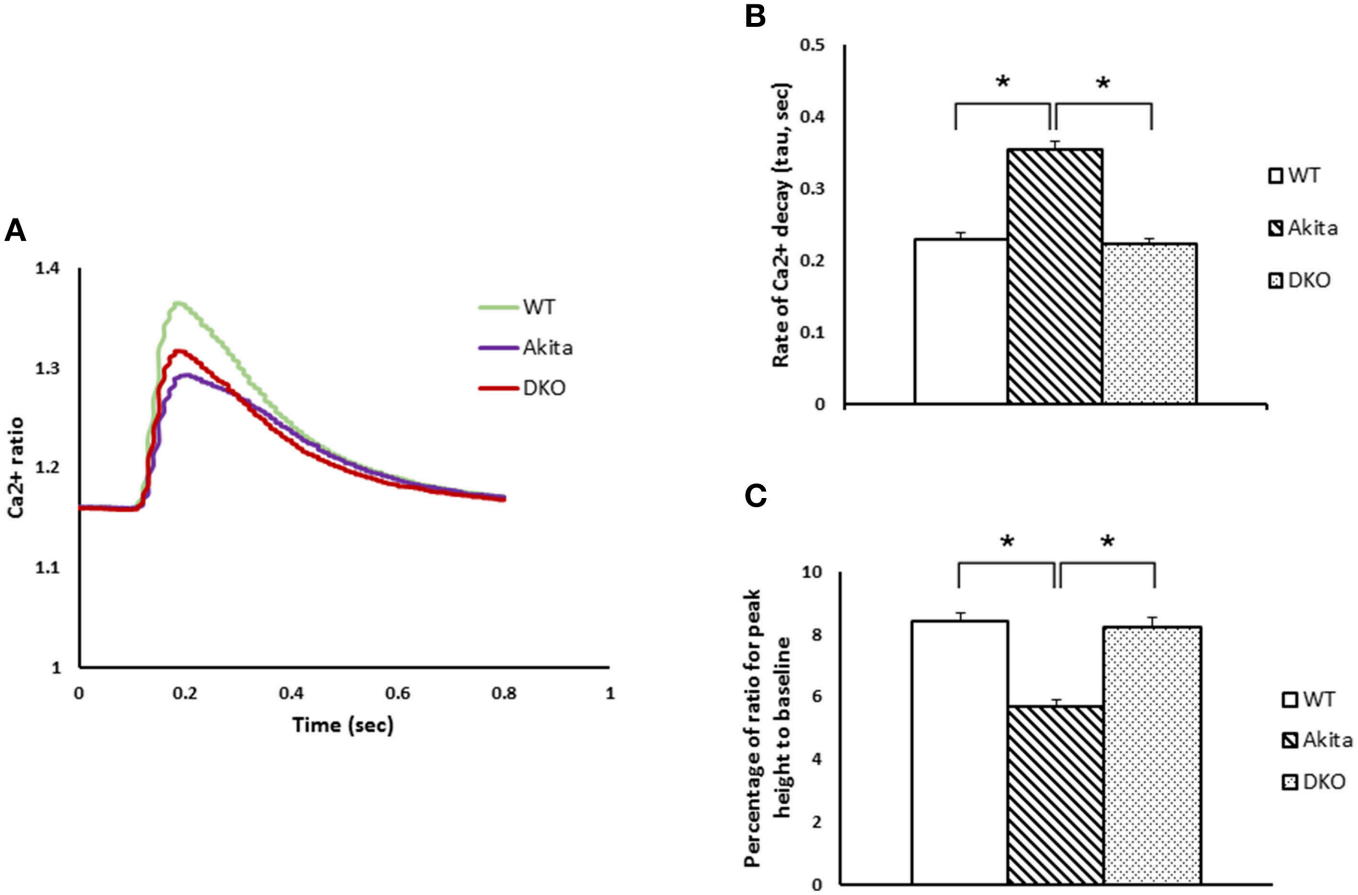
**Abrogation of MMP9 improves cardiomyocytes calcium transients in diabetic Akita mice**. **(A)** Fluorescence ratio of FURA-2 binding to calcium was recorded from myocytes isolated from WT, Akita, and DKO hearts. Cardiomyocytes were stimulated at 1 Hz and calcium ratio was measured during contraction/relaxation cycle. **(B)** Rate of calcium decay per second in WT, Akita, and DKO cardiomyocytes showing increased decay in Akita and restoration of decay in DKO. **(C)** Calcium released during contraction-relaxation cycle was decreased in Akita but normalized in DKO. Each bar represents mean ± SEM from 15 to 20 cardiomyocytes from each group. *N* = 4–7. ^*^p < 0.05.

Calcium transients have important role in regulating contractility of cardiomyocytes (Boudina and Abel, 2007). Therefore, we evaluated calcium transients in WT, Akita, and DKO cardiomyocytes during contraction-relaxation cycle using Fura-2 AM calcium detection dye. Our results showed that calcium transients decreased in Akita as compared to WT but improved in DKO cardiomyocytes (Figure 2A). To determine if decreased calcium transient was due to increased calcium decay, we measured the rate of calcium decay in all the three groups. As expected, the rate of calcium decay was increased in Akita but restored in DKO cardiomyocytes (Figure 2B). Further, we measured percentage of calcium ratio for peak height to baseline, which was decreased in Akita and normalized in DKO cardiomyocytes (Figure 2C). These results demonstrated that calcium transient was decreased in Akita and improved in DKO cardiomyocytes suggesting that inhibition of MMP9 improves calcium transients in cardiomyocytes from diabetic hearts.

### Deletion of MMP9 drives elevation in SERCA-2a expression in cardiomyocytes

**Figure 3:**
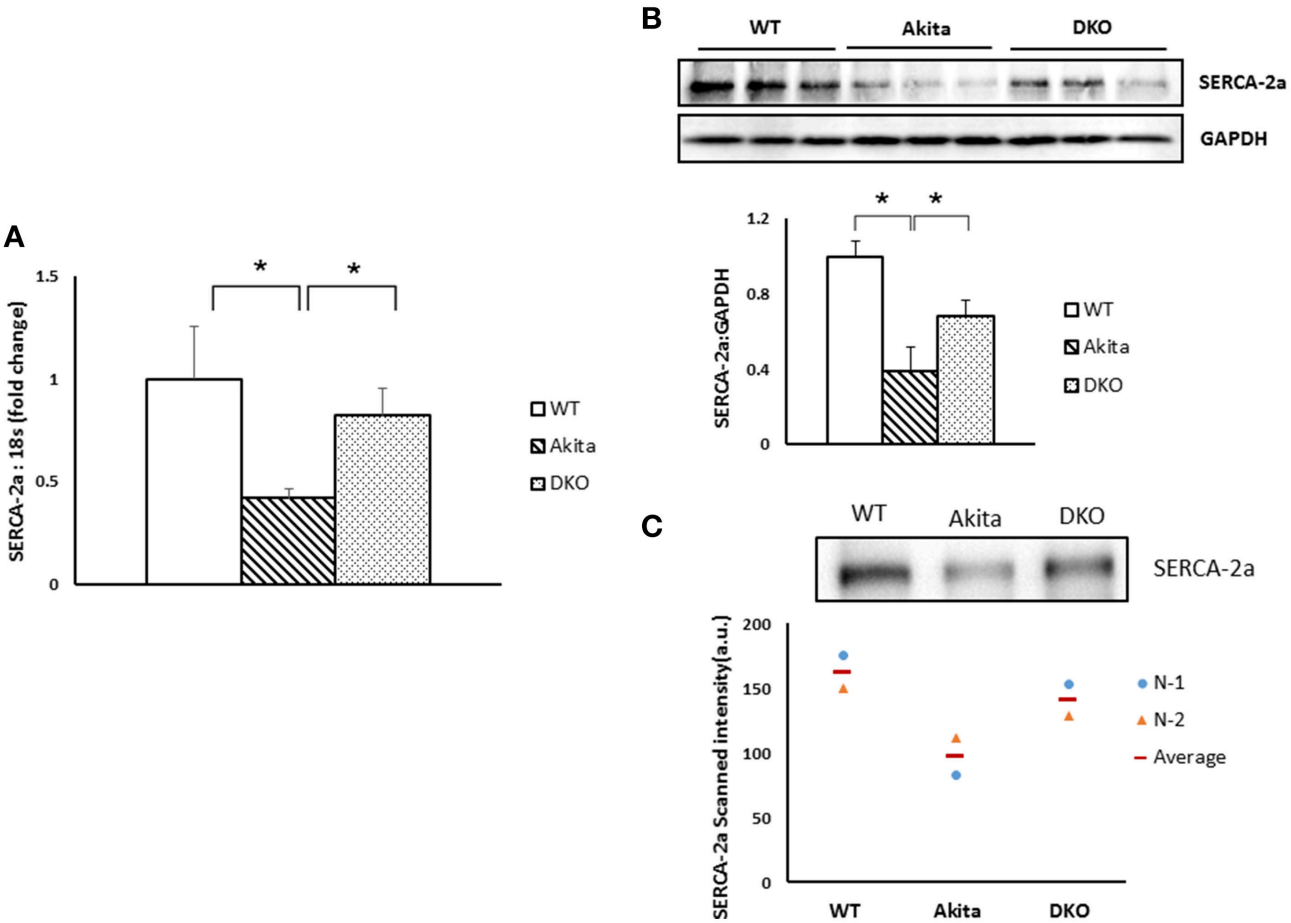
**Deletion of MMP9 gene increased the levels of SERCA-2a in diabetic hearts**. **(A)** QPCR showing the mRNA levels of SERCA-2a in WT, Akita, and DKO hearts. Values are mean ± SEM. *N* = 5, ^*^P < 0.05. **(B)** Top, representative Western blot bands for SERCA-2a and GAPDH (a loading control). Bottom, bar graph showing densitometric analyses of SERCA-2a band intensity. *N* = 3. Values are mean ± SEM. ^*^P < 0.05. **(C)** Top, representative immunoprecipitation bands for SERCA-2a. Bar graphs showing relative expression of SERCA-2a in the three groups. Bottom, bar graph showing the relative values of densitometric band intensity (arbitrary unit, a. u.) *N* = 2.

SERCA-2a is one of the important calcium handling proteins in cardiomyocytes and it is downregulated in diabetic hearts (Zarain-Herzberg et al., 2014). To determine if reduced level of SERCA-2a contributed to decreased calcium transients in Akita and abrogation of MMP9 improved calcium transients by upregulating SERCA-2a, we measured the mRNA and protein levels of SERCA-2a in WT, Akita and DKO hearts. QPCR results showed that SERCA-2a mRNA level was decreased in Akita as compared to WT hearts. However, it was significantly increased in DKO as compared to Akita (Figure 3A). Western blotting and immunoprecipitation results demonstrated that SECRA-2a protein expression was decreased in Akita and increased in DKO hearts (Figures 3B,C). These results suggest that inhibiting of MMP9 improves SERCA-2a expression in diabetic hearts.

Altogether, our results revealed that ablation of MMP9 improves contractility and calcium transients of cardiomyocytes, plausibly by improving SERCA-2a in diabetic hearts.

## Discussion

**Figure 4:**
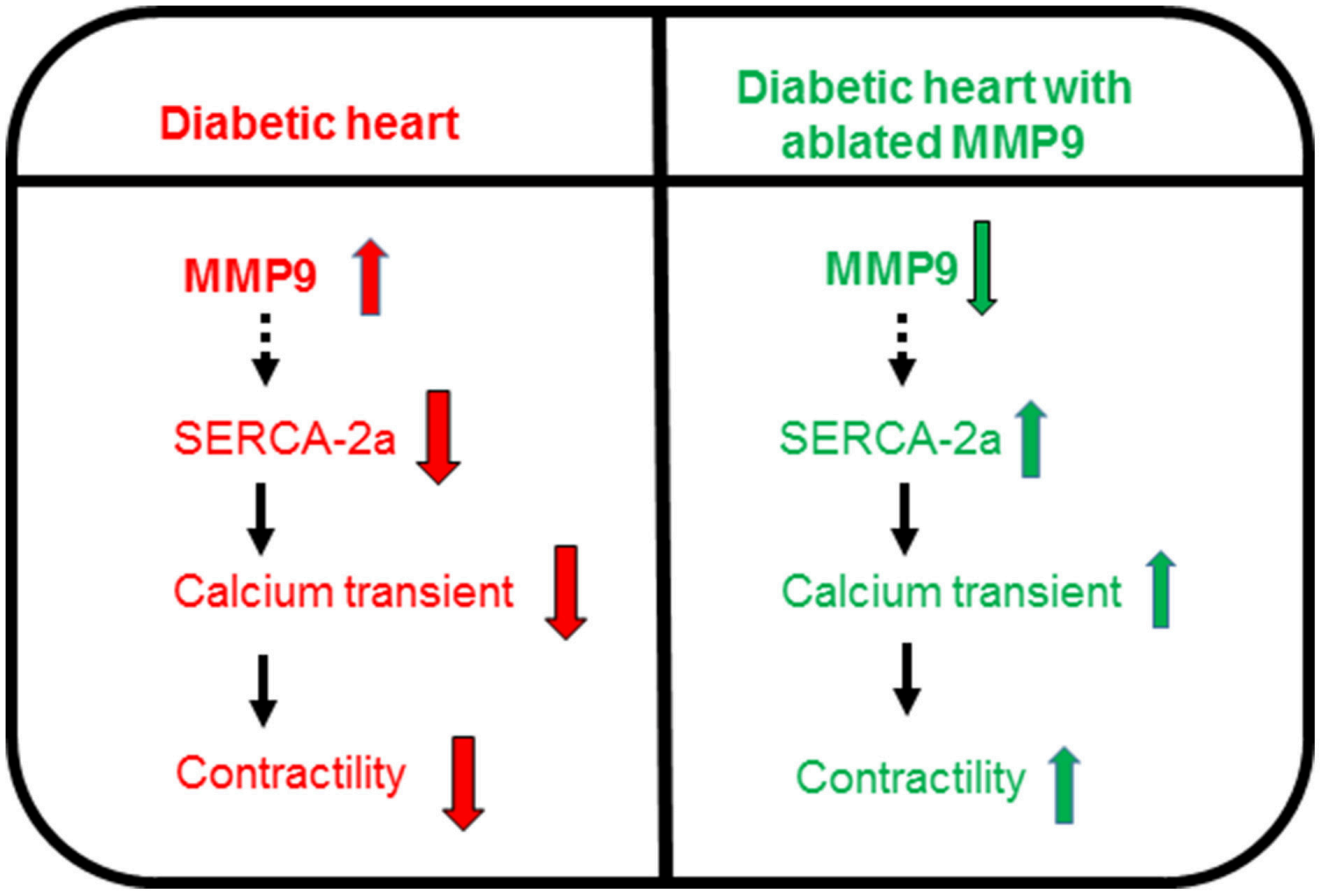
**Schematic showing increased activity of MMP9 in diabetic hearts is involved in downregulation of SERCA-2a that decreases calcium transients and impairs contractility of cardiomyocytes**. However, ablation of MMP9 nullifies downregulation of SERCA-2a, improves calcium transients, and increases contractility of cardiomyocytes in diabetic hearts.

We observed that in DKO mice, SERCA-2a is upregulated in the heart, calcium transient is improved, and contractility is increased in cardiomyocytes as compared to diabetic Akita (Figures 1–3). In Akita hearts MMP9 is upregulated (Mishra et al., 2010b) whereas in DKO hearts MMP9 is abrogated (Mishra et al., 2012), therefore, improvement in contractility in DKO is plausibly due to ablation of MMP9 gene. We propose that activation of MMP9 downregulates SERCA-2a, impairs calcium transients, and contractility of cardiomyocytes, whereas abrogation of MMP9 upregulates SERCA-2a, improves calcium transients, and contractility of cardiomyocytes in diabetic hearts (Figure 4). These results support our hypothesis that MMP9 regulates contractility of cardiomyocytes in diabetic hearts and ablation of MMP9 prevents cardiomyocytes contractile dysfunction in diabetics. Considering impaired cardiac contractility as a major cause of diabetic cardiomyopathy (Malhotra and Sanghi, 1997; Boudina and Abel, 2007), our results provide a novel role of MMP9 in diabetic hearts and a novel therapeutic approach for ameliorating contractile dysfunction of diabetic hearts by suppressing MMP9.

MMP9 is elevated in diabetic (Tayebjee et al., 2004; Mishra et al., 2010b) and failing (Mann and Spinale, 1998; Thomas et al., 1998; Buralli et al., 2010; Chu et al., 2013) hearts. Increased plasma levels of MMP9 is associated with mortality in humans (Buralli et al., 2010) and with pathological cardiac remodeling (Halade et al., 2013). We have previously reported that in cardiomyocytes from C57BL/6J mice, addition of MMP9 protein decreases contractility whereas pre-treatment with MMP9 inhibitor TIMP4 on cardiomyocytes blunts the effect of MMP9 on contractility (Mishra et al., 2010c). However, the mechanism of MMP9-mediated contractile dysfunction was unclear. It is reported that plasma MMP9 cleaves extracellular domain of β2-adrenergic receptors (Rodrigues et al., 2010) that induce contractility of cardiomyocytes. Conversely, pharmacological targeting of β-adrenergic functions abrogates MMP9 secretion in medulloblastoma cells (Annabi et al., 2010) suggesting a potential cross-talk between β-adrenergic receptors and MMP9 (Rietz and Spiers, 2012). The β-adrenergic receptors induces SERCA-2a at downstream to increase contractility of cardiomyocytes. Therefore, inhibition of β-adrenergic receptors signaling suppresses SERCA-2a activity. Ours results demonstrate that in Akita where MMP9 is activated (Mishra et al., 2010b) SERCA-2a level is low and in DKO where MMP9 is abrogated SERCA-2a level is high (Figure 3). It suggests that MMP9 is involved in regulation of SERCA-2a in diabetic hearts. Further, it supports the previous reports that MMP9 plays an important role in contractility of cardiomyocytes.

Although ablation of MMP9 upregulates SERCA-2a in diabetic hearts, it is unclear whether MMP9 acts through β-adrenergic receptors at extracellular level, or it directly regulates SERCA-2a at intracellular level. Previously, it is reported that mitochondrial MMP9 alters calcium homeostasis and increases mitochondrial pore transition leading to contractile dysfunction in hyperhomocysteinemic cardiomyocytes (Moshal et al., 2008a). It suggests a possible role of MMP9 in calcium signaling-mediated impairment of SERCA-2a activity in diabetic hearts. However, more studies are required to confirm it. The role of MMP9 in regulation of SERCA-2a is also an important finding because SERCA-2a overexpression improves contractility of cardiomyocytes obtained from failing human hearts (del Monte et al., 1999). The improvement in contractility of cardiomyocytes concomitant with increase in SERCA-2a levels in DKO heart (Figures 3A–C) supports previous studies showing that SERCA-2a upregulation mitigates contractile dysfunction of cardiomyocytes (Giordano et al., 1997; He et al., 1997; del Monte et al., 1999; Karakikes et al., 2009; Cutler et al., 2012). Our results also support the cardioprotective effect of inhibition of MMP9 in pathological hearts (Romanic et al., 2002; Lindsey et al., 2006; Moshal et al., 2008b; Chiao et al., 2012).

We are intrigued to observe that rate of calcium decay (Figure 2B) and percentage of calcium ratio for peak height to baseline (Figure 2C) is restored and not blunted in DKO. This finding supports the crucial role of MMP9 in calcium homeostasis (Moshal et al., 2008a). Moreover, both rate of contraction (Figure 1B) and rate of relaxation (Figure 1C) are blunted in DKO as compared to Akita suggesting that MMP9 in involved in regulation of both systolic and diastolic dysfunction. Diabetic hearts at early stage show only diastolic dysfunction and in later stage progress to systolic dysfunction (Boudina and Abel, 2007). In 12–14 week male Akita show both diastolic and systolic dysfunction, which is mitigated by ablation of MMP9 (Figures 1B,C). Therefore, we presume that abrogation of MMP9 may have potential effects on contractility of diabetic hearts with diastolic dysfunction.

Altogether, these results provide a novel insight on MMP9-mediated contractile dysfunction in diabetics and opens a new window for future studies for cardiac specific role of MMP9 on contractility in diabetic hearts.

## Author contributions

PP wrote the manuscript and contributed in data generation, NM and SN contributed in data generation, ST contributed in drafting the manuscript, PM conceptualized the idea, contributed in generating data, and drafting the manuscript.

## Funding

This work was supported, in part, by the National Institutes of Health grants HL-113281 and HL-116205 to PM, and HL-74185 to ST.

### Conflict of interest statement

The authors declare that the research was conducted in the absence of any commercial or financial relationships that could be construed as a potential conflict of interest.
